# Ultra-Bend-Resistant 4-Core Simplex Cable Used for Short-Reach Dense Spatial Division Multiplexing Optical Transmission

**DOI:** 10.3390/mi15010108

**Published:** 2024-01-07

**Authors:** Zelin Zhang, Yu Qin, Jie Zhu, Caoyuan Wang, Xinli Jiang, Yichun Shen, Limin Xiao

**Affiliations:** 1 Advanced Fiber Devices and Systems Group, Key Laboratory of Micro and Nano Photonic Structures (MoE), Key Laboratory for Information Science of Electromagnetic Waves (MoE), Shanghai Engineering Research Center of Ultra-Precision Optical Manufacturing, School of Information Science and Technology, Fudan University, Shanghai 200433, China; 2 Zhongtian Technology Group, Nantong 226463, China

**Keywords:** multicore fiber, optic cable, macro-bending loss, cross-talk, eye diagram

## Abstract

We optimized and fabricated an ultra-bend-resistant 4-core simplex cable (SXC) employing 4-core multicore fiber (MCF) suitable for short-reach dense spatial division multiplexing (DSDM) optical transmission in the O-band. The characteristics of transmission loss, macro-bending and cross-talk (XT) between adjacent cores after cabling were firstly clarified. By introducing the trapezoid index and optimizing the cabling process, the maximum values of added XT of 1.17 dB/km due to 10 loops with a bending radius of 6 mm imposed over the 4-core SXC and a macro-bending loss of 0.37 dB/10 turns were, respectively, achieved.P Then, the optical transmission with low bit error rate (BER) was presented using a 100GBASE-LR4 transceiver over the 1.2 km long 4-core SXC. The excellent bending resistance of the 4-core SXC may pave the way for a reduction in space pressure and increase in access density on short-reach optical interconnect (OI) based on DSDM.

## 1. Introduction

The demands for the interconnected data-center (DC) have been experiencing an urgent growth of larger bandwidth (BW) and higher optical access density [1]. Thus, many new special cables and connecting technologies have been proposed for short-reach optical interconnects (OI) between DC buildings or floors, such as reduced-coating high-density cable, spiderweb ribbon cable, spatial multiplexing division (SDM) technology and so on [2,3,4,5,6]. In particular, compared with the conventional single-core single-mode fiber (SC-SMF), the SDM technology over MCFs containing several fiber cores within common cladding enables the MCF cable to be applied to overcome the physical-space constraints of the pathway [7]. To date, a 1152-core outdoor cable suitable for a metro network, a 2304-core outdoor cable employing a 200 μm 4-core fiber and a 96-core outdoor cable using an 8-core fiber have been reported to achieve a breakthrough in capacity limits in the existing optical fiber communication infrastructure [1,3,7]. However, these methods need to use numerous SC-SMF SXCs or ribbon cables to achieve the OI between thousands of conventional optical modules and fan-in/fan-out (FIFO) devices, which will cause space congestion in DC [1,7,8]. By using an MCF-SXC and combining it with an MCF connector and MCF optical transceiver [9,10,11,12,13,14,15], it can provide an optimal solution to further release the space pressure, save power consumption and increase the access density on OI in DC [12,13]. But there are few studies on the manufacture process and transmission characteristics of the MCF-SXC.

In our work, by optimizing refractive index profile (RIP) design, drawing and cabling processes, we fabricated an optimized ultra-bend-resistant 4-core SXC employing a 4-core MCF in the O-band used for short-reach DSDM optical transmission. Then, the characteristics of transmission loss, macro-bending loss and XT between adjacent cores due to the cabling were firstly presented. These experimental results show that the 4-core SXC has an excellent bending resistance for short-reach OI in narrow space. Finally, the optical transmission with low BER in the O-band was demonstrated using a 100GBASE-LR4 (Zhongtian Technology Group, Nantong, China) transceiver over a 1.2 km long 4-core SXC. These experimental results indicate that the prepared ultra-bend-resistant 4-core SXC would have potential applications to further release the space pressure, reduce power consumption and improve the transmission capacity on short-reach DSDM optical transmission.

## 2. Fabrication of the Ultra-Bend-Resistant 4-Core SXC

**Figure 1:**
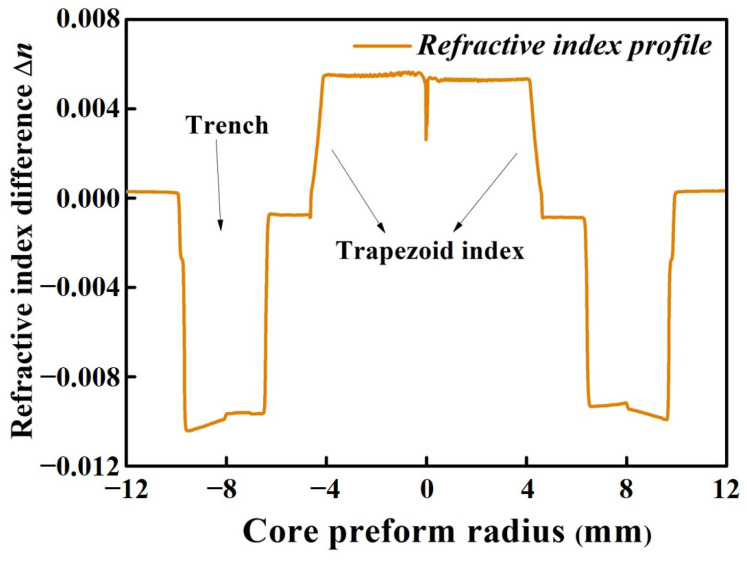
Measured refractive index profile of core preform.

**Figure 2:**
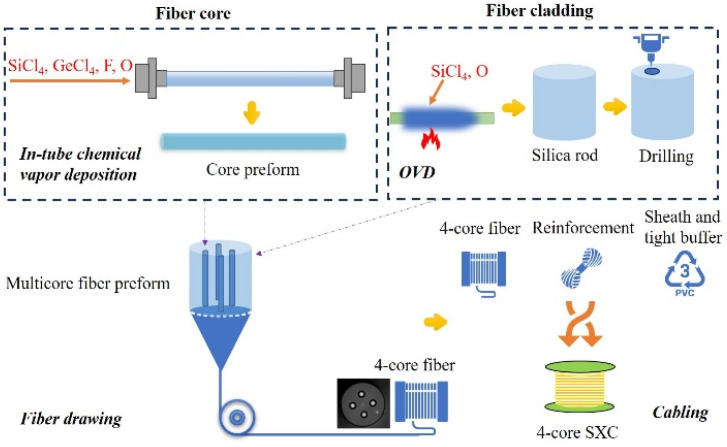
Whole fabrication process flow of the ultra-bend-resistant 4-core SXC.

The XT between adjacent cores and macro-bending loss in multicore fiber resulting in the degradation of signal quality can be suppressed by adjusting the RIP design [1,16]. In our work, by introducing the trapezoid index and deep assisted trench around the central core, the 4-core fiber was designed to keep a low XT and low macro-bending loss for transmission in the O-band [17]. Figure 1 shows the measured RIP of core preform by using PK2600 (Photon Kinetics, Beaverton, OR, USA). The whole fabrication process flow of the ultra-bend-resistant 4-core SXC can be orderly divided into three parts: preform deposition, fiber drawing and cabling, which are, respectively exhibited in Figure 2. For the preform deposition, in order to avoid the OH-absorption during the manufacture process of the preform as much as possible [18], we used the in-tube chemical vapor deposition with a deep fluorine-doped process for the wave-guided core and the OVD process for the high-purity silica rod, respectively. The original diameter and length of the core preform are 24.89 mm and more than 950 mm, respectively. By using the fine extending and corrosion processes, the outer diameter of the core preform was changed around 15.90 mm to match with the target inner diameter of the drilling hole. Then, based on a high-precision drilling process, the core preforms were slit and inserted into a silica rod to assemble the multicore fiber preform. The final outer diameter and length of the 4-core fiber preform were 80.02 mm and 300 mm, respectively. Compared with the Stack-and-Draw manufacture process of MCF [19], our Drilling-and-Draw manufacture process aims to further improve the geometric accuracy of the core layer in MCF, which may reduce the mismatch with fan-in/fan-out (FIFO) devices. In the experiment, by using the in-line fiber drawing process, the 4-core fiber preform was drawn into fiber by optimizing a lower heating temperature around 1650 °C and higher drawing tension (100 ± 3 g), so as to keep a better geometrical uniformity. The silica fiber was coated by using UV radiation-curable acrylate material as a flexible protective layer, and the effective drawn length of the 4-core fiber was more than 92 km.

**Figure 3:**
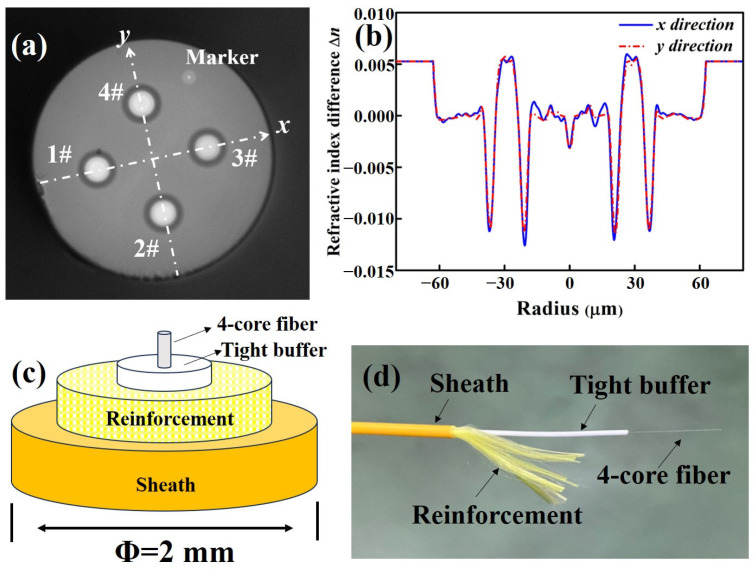
Fabricated 4-core fiber and 4-core SXC. (**a**) Cross-section of the 4-core fiber, (**b**) measured RIP of the 4-core fiber, (**c**) schematic view of the structure of the 4-core SXC, and (**d**) external view of the 4-core SXC.

The cross-section of the 4-core fiber is shown in Figure 3a, where the core pitch, outer diameters of cladding and coating are 41.2 ± 0.2 μm, 124.5 ± 0.6 μm and 245.0 ± 1.0 μm, respectively. Figure 3b shows the measured RIP of the 4-core fiber by using IFA-100 (Interferometric Fiber Analyser, Sharon, MA, USA). Limited by the measured position resolution, the measured RIPs of the 4-core fiber along the *x* and *y* directions are different from those of the core preforms. It can be found that the index depth of the assisted trench is closed to −12.0 × 10^−3^ after fiber drawing, which can confine the guided optical wave within the core as much as possible. Two improvements of enhancing the mechanical robustness of 4-core SXC were carried out during the cabling process. One improvement is that the tensile strength of the 4-core fiber was increased to 107 kpsi which is 7% higher than that of SC-SMF. Another improvement is using aramid yarn mixed with polyester yarn and modified PVC as the reinforcement and sheath of the 4-core SXC, ensuring that the elongation at break and the tensile strength of the 4-core SXC can reach up to 180% and 15 MPa, respectively. Figure 3c,d show the schematic and external views of the structure of the fabricated 4-core SXC, where the outer diameters of the tight buffer and sheath are 0.85 ± 0.05 mm and 2.0 ± 0.1 mm, respectively.

## 3. Transmission Characteristics of Ultra-Bend-Resistant 4-Core SXC

As shown in Figure 1 and Figure 3, the measured RIP results and geometrical structure of the fabricated 4-core SXC can remain greatly consistent with the expected design. Then, we will test the transmission characteristics of this 4-core SXC and demonstrate its potential optimization of fabrication process in the future.

### 3.1. Transmission Loss

**Figure 4:**
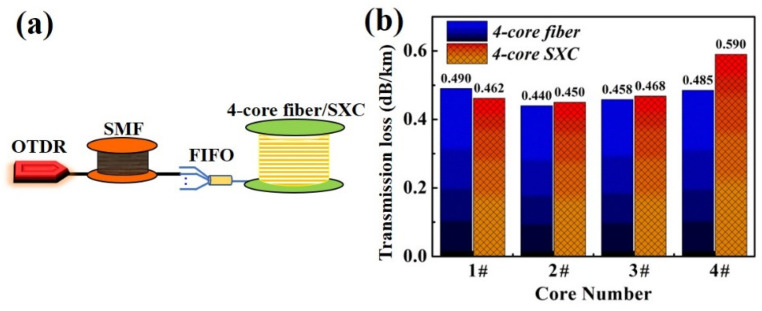
Transmission loss test. (**a**) Experimental setup by OTDR and (**b**) test results.

In this section, we will show the new finding of transmission loss after the cabling process on the 4-core SXC. Firstly, we measured the mode field diameters (MFD) of these four cores which are approximately 8.4 μm at 1310 nm. And the measured values of cutoff wavelength (from 1# to 4#) are 1207.8 nm, 1213.8 nm, 1228.9 nm and 1216.5 nm, respectively. The transmission loss of each channel in the O-band was measured by the prepared tapered-type FIFO device and optical time domain reflectometry (OTDR), which are shown in Figure 4a. The duration time of probe pulse and average time for OTDR were adjusted to 100 ns and 30 s, respectively. In our experiment, the maximal insertion loss and average XT between adjacent channels of the FIFO device are, respectively, less than 0.8 dB and −70 dB at 1310 nm, which can achieve a high coupling efficiency with the fabricated 4-core SXC [20]. By using the fusion splicer for the core alignment of the rotational direction and controlling the cleaved angle (<0.5°), the fusion splice loss between the tail fiber of the FIFO device and the 4-core fiber/SXC can be less than 0.2 dB. The 2.9 km long standard single-mode fiber (SMF) was firstly placed to remove the blind zone of OTDR. Figure 4b exhibits the transmission loss results of the 4-core fiber and 4-core SXC, respectively. It can be found that the transmission loss of all cores before cabling is less than 0.500 dB/km at 1310 nm. It is analyzed that the transmission loss of our prepared 4-core fiber can be mainly divided into four parts: the intrinsic infrared absorption of silica (SiO_2_) material and residual OH- (*α_i_*), the Rayleigh scattering caused by microscopic density fluctuation (*α_R_*), the micro-bending loss induced by small-scale bends from fiber coating (*α_b_*) and the potential coating-leakage loss (*α_l_*). In the future, by adjusting the GeO_2_ dopant, further reducing the OH- content and optimizing the process of fiber coating, the intrinsic transmission loss of the 4-core fiber might be below 0.37 dB/km in the O-band [1]. Moreover, there are no significant changes in transmission loss of the 1#, 2# and 3# core after cabling, as shown in Figure 4. However, the transmission loss of the 4# core shows an obvious degradation of 0.105 dB/km due to the cabling. This can be explained by the fact that the 4# core which is the nearest neighbor to the marker core would suffer most from the geometric deformation of the core and the longitudinal structural fluctuation when the 4-core fiber is cabled. It would cause the micro-bending loss due to fiber compression in the longitudinal direction of the 4-core SXC [3,16,17]. Fortunately, finer control over a tight construction or adjusting the buffer coating material can be used to minimize this cabled loss degradation (<0.010 dB/km) [1,21]. 

### 3.2. Macro-Bending Loss

**Figure 5:**
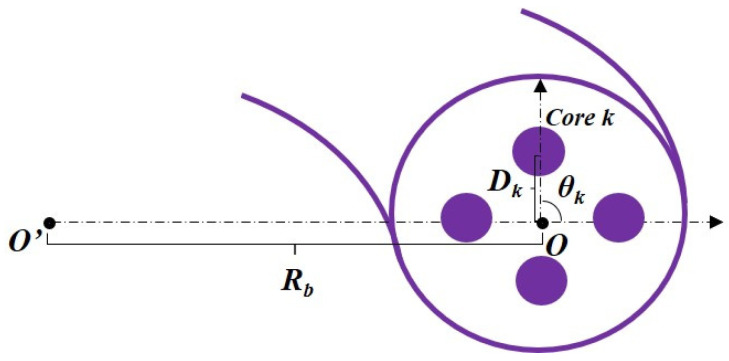
Simple description of the local polar coordinate in a bent 4-core fiber.

The macro-bending loss is one of the most significant characteristics of the multicore fiber cable used for short-reach OI in narrow space. The macro-bending loss (*α_BL_*) associated with the imaginary part of effective refractive index (*n_eff_*) can be expressed as [22]:

(1) $$\alpha_{BL}=\frac{20}{\ln(10)}\frac{2\pi }{\lambda }imag(n_{eff})$$

where *λ* is the transmission wavelength. By using the equivalent index model [23], the effective refractive index of core *k* (*n_eff_k_*) in a bent 4-core fiber can be described as [23]:

(2) $$n_{eq}\approx n_{eff\_k}(1+\frac{D_{k}\cos\theta_{k}}{R_{b}}),\;\frac{D_{k}\cos\theta_{k}}{R_{b}}<<1$$

where the center *O* of 4-core fiber is the origin of the local polar coordinate shown in Figure 5. *R_b_* and *n_eq_* are the bending radius and equivalent refractive index of the bent core *k*, respectively. *θ_k_* = 0 in the radial direction of the bend. As shown in Equation (2), the macro-bending loss of the fiber cores will have differences due to the different positions of the cores.

In order to experimentally investigate the bending-resistant performance of the 4-core SXC at 1310 nm, 10 loops were imposed with a bending radius of 6.0, 8.2, 10.0 and 18.3 mm over the 4-core SXC and the measured results are shown in Table 1. It can be seen that the maximum value of macro-bending loss with a minimal bending radius of 6.0 mm is only 0.37 dB/10 turns and the average value is only 0.29 dB/10 turns. When the values of the bending radius (*R_b_*) are 8.2 mm, 10.0 mm and 18.3 mm, the corresponding maximum values of measured macro-bending loss are less than 0.10 dB/10 turns, 0.04 dB/10 turns and 0.02 dB/10 turns, respectively. The excellent bending resistance of the 4-core SXC can be explained by inducing the deep F-doped trench-index profile and controlling the MFD of the 4-core fiber. Taking the case with bending radius of 18.3 mm as an example, by using the finite element analysis (FEA) method and solving Equations (1) and (2), the calculated values of the equivalent refractive index for the four cores are 1.4461, 1.4485, 1.4508 and 1.4485 at 1310 nm, respectively. And the corresponding values of macro-bending loss can be calculated as 6.4 × 10^−4^ dB/10 turns, 1.1 × 10^−3^ dB/10 turns, 9.7 × 10^−4^ dB/10 turns and 1.1 × 10^−3^ dB/10 turns, respectively. Actually, the torsional effect which can also induce the additional loss is inevitable in our measurement, which may mainly produce larger measured values than the theoretical values for the 4-core SXC. Perhaps, the combination of the refractive index trench and air holes around the fiber cores would be utilized to further suppress the macro-bending loss in the O-band [22].

### 3.3. XT between Adjacent Cores

In this section, we will investigate the *XT* between adjacent cores due to the cabling and bending dependence of *XT*. For the weakly coupled multicore fiber, the *XT* between adjacent cores accumulated along fiber length *L* can be evaluated by [1,16]

(3) XT≈h⋅L

where *h* is the power-coupling coefficient between adjacent cores, which can be approximately expressed as [1,16]

(4) $$h\approx \kappa^{2}\frac{\lambda R_{b}}{\pi n_{b}\Lambda_{km}}$$

where *κ* and *λ* are the mode-coupling coefficient and transmission wavelength, respectively. *n_b_* is the average equivalent refractive index of the bent core. *Λ_km_* is the distance between adjacent core *m* and core *k*. 

**Figure 6:**
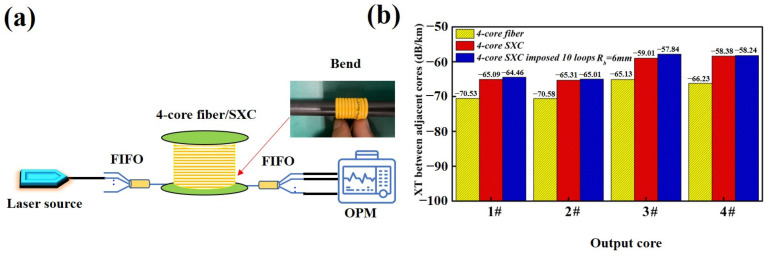
XT between adjacent cores test. (**a**) Experimental setup and (**b**) test results.

As shown in Equation (4), the XT level will be directly affected by the bending radius *R_b_*. Thus, the XT between adjacent cores under bending conditions was measured and the experimental setup is shown in Figure 6a. The laser source is a 1310 nm single-wavelength fiber laser. Its output power is −7 dBm and the power fluctuation is less than 0.01 dB. The optical power meter (OPM) with four channels can be used to monitor the output power from the 4-core fiber and SXC. In order to investigate the bending dependence of XT sufficiently, 10 loops with a minimal bending radius of 6 mm were imposed on the 4-core SXC. Figure 6b shows the XT characteristics of the 4-core fiber, 4-core SXC and 4-core SXC under bending conditions, respectively. It can be calculated that the average value of the XT between adjacent cores of this trapezoid-index 4-core fiber is −68.12 dB/km, which is 1.12 dB/km lower than that of the step-index core [3,17]. In our experiment, the core pitch (*Λ_km_*) of the prepared 4-core fiber is 41.2 μm. Based on Equations (3) and (4), we can increase the core pitch up to 43 μm, and thus further suppress the intrinsic XT of the 4-core SXC to or below −70 dB/km. In this case, the width and depth of the refractive index trench should be wider and deeper so as to suppress the coating-leakage loss in the O-band due to a shorter core to coating distance (the minimum distance from one center to the coating) [1]. As shown in Figure 6b, the average XT between adjacent cores of the 4-core SXC and 4-core SXC with a minimal bending radius can be calculated as −61.95 dB/km and −61.39 dB/km, respectively. The XT changes from the non-cabled 4-core fiber originate from the micro-bend-affected crosstalk induced by the actual cabling processes and winding on the bobbin with the radius of 15.4 cm as well as the longitudinal structural fluctuation [1,16], especially the 3# and 4# cores which are, respectively, the first and second nearest neighbors to the marker core. It can be found that the maximum value and average value of the XT degradation induced by the bending are only 1.17 dB/km and 0.56 dB/km, respectively. These measured results indicate that the prepared 4-core SXC with an excellent bending resistance will have a considerable application prospect on SDM optical transmission in narrow space.

## 4. Signal Transmission of Ultra-Bend-Resistant 4-Core SXC

**Figure 7:**
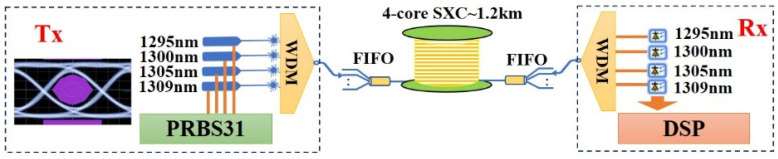
A schematic diagram for the signal transmission experiment.

Finally, the signal transmission experiments of the 4-core SXC link were conducted by using a 100GBASE-LR4 transceiver as the transmitter (Tx) and receiver (Rx). Figure 7 is the schematic diagram of the signal transmission system. In the Tx, four channels satisfying the Lan Wavelength Division Multiplexing (LWDM) standard were excited, where the central wavelengths (*λ*) are around 1295 nm, 1300 nm, 1305 nm and 1309 nm, respectively [24]. By using the direct modulation method, the laser diode was modulated by a pseudo-random binary sequence (PRBS31) with 2^31^-1 bits (25 Gb/s/*λ*). The signal propagated through the fan-in device was launched into the 1.2 km long 4-core SXC. After passing through the fan-out device, the signal was received by the corresponding photo detectors (PD) in Rx. By properly choosing the FIFO device ports and central wavelength of incident light, the eye diagram and bit error rate (BER) performance of each core at different wavelengths over the 4-core SXC were both obtained. The inset figure in Figure 7 is the eye diagram of the back-to-back transmitted signal.

**Figure 8:**
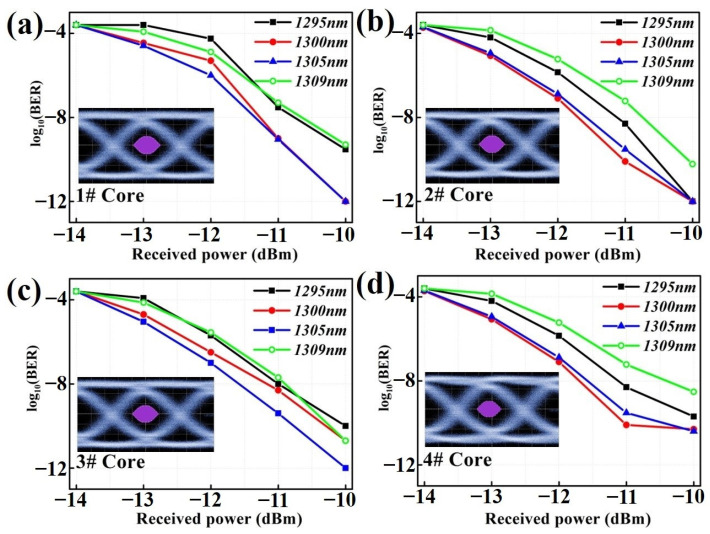
BER performance and eye diagram over the 1.2 km-long 4-core SXC of the (**a**) 1# core, (**b**) 2# core, (**c**) 3# core, and (**d**) 4# core.

When the signal light is injected into different cores, the measured BER curves at four wavelengths are shown in Figure 8. It can be seen that the 4# core, which is the nearest neighbor to the marker core, shows a relatively larger deterioration of the BER curve. Based on the above-mentioned experimental results, the higher XT level and larger transmission loss degradation due to cabling would cause this deterioration of the BER curve. Additionally, the changes of polarization-mode dispersion (PMD) inside the fabricated 4-core SXC should not be neglected, which could be attributed to the differences in the intrinsic birefringence parameters of each core. Significantly, the 4# core which is the first nearest neighbor to the marker core would suffer from a severer PMD due to a larger core ellipticity that can also lead to this deterioration of the BER curve [25]. In the future, the optimization of the fiber drawing and cabling process around the marker core can provide a feasible and reliable way to improve the BER performance of the 4-core SXC. 

Furthermore, the inset figures in Figure 8 are the eye diagrams with the most obvious deteriorations of the BER curve, which are 1295 nm of the 1# core, 1309 nm of the 2# core, 1295 nm of the 3# core and 1309 nm of the 4# core, respectively. Compared with the eye diagram of the back-to-back case, there are no significant distortions in the eye diagrams and they are clearly open after propagating through the 1.2 km long 4-core SXC. Actually, the used length of an indoor SXC in terminated cable assemblies generally varies from several to tens of meters. Thus, the impacts caused by XT and transmission loss will be further reduced due to the shorter transmission distance in short-reach OI. It is worth noting that, compared with the conventional SC-SXC and duplex cable, this 4-core SXC can, respectively, obtain 75% and 50% space reduction in existing short-reach OI infrastructure, which can achieve the hybrid unidirectional and bi-directional transmission by using the 4-core SXC. In this case, the induced propagation constant mismatch due to co-propagation will further suppress the XT level in the SDM transmission system [26,27,28]. Additionally, once the fabricated 4-core SXC with a tight design is installed in practice, the environmental effects, such as temperature, moisture and mouse biting, would have significant impacts on transmission performance [21,29]. For example, the polymer materials used as the different layers of the 4-core SXC have individual coefficients which are quite different from that of the silica fiber. During the outdoor service of the wiring closet, the differential expansion and contraction can induce temperature-dependent macro-bending and micro-bending loss. In the future, the combination of a soft buffer and hard jacket or silicone buffer would ensure better performance at low temperatures for the 4-core SXC, and the armored technology will also be worth in-depth study [21,29]. Moreover, the aramid yarn mixed with polyester yarn, which was used as reinforcement in our experiment, is not able to block water, which will accelerate the crack growth rate and hydrogen loss in a humid environment. Filling with water blocking jelly and using a fiber coating free of hydrogen may prolong the cable lifetime and minimize the fiber loss that is susceptible to hydrogen [29].

## 5. Conclusions

In conclusion, we designed and fabricated an ultra-bend-resistant 4-core SXC in the O-band used for short-reach DSDM optical transmission. We experimentally clarified the characteristics of transmission loss, macro-bending and XT between adjacent cores due to the cabling, respectively. The ultralow macro-bending loss and small XT degradation induced by the bending indicate that the prepared 4-core SXC has an excellent bending resistance. Then, we demonstrated a low BER signal transmission by using a 100GBASE-LR4 transceiver. By optimizing the fiber fabrication and cabling process, the transmission loss, XT and BER performance will be further improved. In the future, combined with an MCF connector, MCF transceiver and low-loss splicing of MCF [9,10,11,12,13,14,15,30,31], the prepared ultra-bend-resistant 4-core SXC would have potential applications to further release the space pressure, reduce power consumption and improve the transmission capacity on short-reach DSDM optical transmission. Furthermore, by using plasmonic material as fiber coating, the 4-core fiber may also be extended in the industry for applications in advanced sensors, surface-enhanced Raman spectroscopy and so on [32,33].

## Figures and Tables

**Table 1:** Measured macro-bending loss at different bending radii.

Bending Radius	Macro-Bending Loss (dB/10 Turns)			
	1#	2#	3#	4#
*R_b_* = 6.0 mm	0.37	0.22	0.37	0.21
*R_b_* = 8.2 mm	<0.10	<0.10	<0.10	<0.10
*R_b_* = 10.0 mm	<0.04	<0.04	<0.04	<0.04
*R_b_* = 18.3 mm	≤0.02	≤0.02	≤0.02	≤0.02

## Data Availability

All the data presented in this study are available in this article.
